# Count Every Newborn: EN-INDEPTH study to improve pregnancy outcome measurement in population-based surveys

**DOI:** 10.1186/s12963-020-00243-y

**Published:** 2021-02-08

**Authors:** Stephen M. Tollman, Peter Byass, Peter Waiswa, Hannah Blencowe, Judith Yargawa, Joy E. Lawn

**Affiliations:** 1 MRC/Wits Rural Public Health and Health Transitions Research Unit (Agincourt), Agincourt, South Africa; 2 Division of Health and Population, University of the Witwatersrand School of Public Health, Johannesburg, South Africa; 3 Department of Epidemiology and Global Health, Umeå University, Umeå, Sweden; 4 Department of Health Policy, Planning and Management, Makerere University School of Public Health, Kampala, Uganda; 5 Centre of Excellence for Maternal Newborn and Child Health Research, Makerere University, Kampala, Uganda; 6 Department of Public Health Sciences, Karolinska Institutet, Stockholm, Sweden; 7 Maternal, Adolescent, Reproductive & Child Health (MARCH) Centre, London School of Hygiene & Tropical Medicine, London, UK

## Every Newborn-INDEPTH study supplement dedication: Professor Peter Byass

**Table:** 

**Professor Peter Byass (1957-2020)** • Professor of Global Health and Director of the Umeå Centre for Global Health Research, Umeå University, Sweden • Chair, Scientific Advisory Committee and Board Member, INDEPTH Network • Chief Editor, Global Health Action • Editorial Board Member, BMC Population Health Metrics • Honorary Professor, University of the Witwatersrand, South Africa • Honorary Professor, University of Aberdeen, Scotland Source: http://www.byass.uk/	

**Dr Tedros Adhanom Ghebreyesus**

*Director General, World Health Organization*

Heartbroken by the passing of Professor @PeterByass, dear friend and mentor to me and countless others around the world. Peter lent his heart and intellect in the service of humanity right up until the end. My deepest condolences to his family. @PeterByass supervised my Ph.D. thesis at the @UniofNottingham and our friendship and collaboration endured. Peter was a staunch advocate for science, equitable solutions and global solidarity, including the #COVID19 pandemic. I pledge to keep his vision alive (@DrTedros, 17th August 2020 tweet).

**Professor Tumani Corrah**

*Board Chair, INDEPTH Network and Director, Africa Research Excellence Fund*

Like a thief, unannounced death stole a colleague to many and a close friend of my family - best described by my son as his surrogate parent. With his demise global health has lost a brilliant mind, an enlightened scholar with the knack to make the complicated simple. Motivated to always think outside the box, Professor Peter Byass was committed to improving health through high quality research. He will be remembered for his dedication to nurturing the next generation of health researchers particularly from LMICs. A founding member of INDEPTH, his significant contributions towards improving the quality of the Network’s science contributed to INDEPTH’s current recognition by the global scientific community. A devout Christian and a member of the clergy, he was wholeheartedly committed to his family and all those fortunate to know him. May his gentle soul rest in perfect peace.

**Professor Stephen M Tollman**

*Director, MRC/Wits University Rural Public Health and Health Transitions Research Unit & Senior External Editor, EN-INDEPTH study supplement*

It is distressing to compose notes of a long-time friend and colleague with whom one has walked a walk of many years. Peter was disarmingly relaxed in manner – yet his thinking, analyses and writing reflect profound insights coupled with deep humanity. It was easy to take his effortless style for granted and as a research partner his gifts, generously shared, helped us all do better. While his research interests were broad, it is Peter’s sustained contributions to understanding mortality and cause of death – and methods to bring this in reach of routine vital registration – that stand out. In recent years his month-long visits to rural South Africa, always with Margaret, allowed frequent forays into the wild, trips that were sacrosanct. His loss, deeply felt, leaves a space that can only be bridged by redoubling our efforts in the fight for life and dignity for all.

**Dr Peter Waiswa**

*For the Makerere University School of Public Health team, Uganda*

Peter so much loved Africa - he pushed for the understanding of the cause of death even where medical data were lacking; he advanced capacity building and was easy to approach, responsive, and a darling to many across HDSS sites. Once approached, Peter was always ready to help. He also made many young Africans become authors by supporting them to publish. His works have led to the saving of many lives of women and children, and his works will live on. He was a true African at heart – and practice. Rest in peace and power Peter.

**Professor Joy E Lawn, Dr Hannah Blencowe and Dr Judith Yargawa**

*For the EN-INDEPTH study supplement editorial team, London School of Hygiene & Tropical Medicine, UK*

Peter Byass worked with us as the Senior Editor on the EN-INDEPTH study supplement over his last year of life. His knowledge of the topic, the teams of the INDEPTH Network and his vast experience as a journal editor were invaluable, including at our last meeting in early August 2020. His death just 2 weeks after this came as a huge shock to us all. He was a delight to work with - thoughtful, kind, and humorous. His passion for empowering researchers from the global south was fundamental. We are deeply privileged to have worked with him and hence have dedicated these 12 papers in the supplement to his memory.

**Dr Nurul Alam**

*For the Matlab HDSS site team, Bangladesh*

I met Professor Peter Byass in INDEPTH Scientific Conference held in Accra in 2005, and subsequently, in workshops led by Peter on analysis of verbal autopsy symptom data using ‘InterVA’ to assign cause of death. I admire his discovery of ‘InterVA.’ Peter visited Dhaka in July 2009 to attend a workshop and I had the opportunity to take him to icddr,b Matlab Health Research Centre and showed him Matlab HDSS field activities. I pray for his eternal peace.

**Dr Yeetey Enuameh**

*For the Kintampo HDSS site team, Ghana*

The Kintampo Health Research Center did not work directly with Professor Peter Byass but felt his influence through the INDEPTH Network. He was the Scientific Committee Chair of the Network and later stepped in as Acting Board Chair when the position became vacant until a substantive Chair was elected. He is credited with and will forever be remembered for his contribution to the development of the InterVA software, that eased the transcription and coding of verbal autopsy interviews by physicians. May he rest in perfect peace.

**Dr Solomon Mekonnen Abebe**

*For the Dabat HDSS site team, Ethiopia*

We are deeply saddened by the sudden passing of Professor Peter Byass. He was not only instrumental in the countless works we were engaged in but his passion stemmed much farther than that. His impact in the lives of Africans will be seen for generations to come and we send our deepest condolences to all of his family and loved ones.

## Why was this study needed?

Each year an estimated 2 million are stillborn in the last three months of pregnancy [1], nearly half dying during labour, while a further 2.4 million babies die in the first 28 days after birth [2], linked to 0.3 million maternal deaths [3]. Millions more children are born too soon, at risk of long-term disabilities [4]. Most of this burden lands on low- and middle-income countries (LMICs). Each of these outcomes are a tragedy for that family, more so as most are preventable. Yet most are still not counted in routine data systems, and the world remains heavily reliant on household survey data to count births, pregnancy outcomes and track maternal and child mortality. The COVID-19 pandemic is disrupting services, increasing stillbirths and neonatal deaths in hospitals [5] and threatening fragile progress to Sustainable Development Goal (SDG) targets for maternal/newborn deaths and stillbirths. Hence, data are even more crucial to protect the most vulnerable populations.

Targets for neonatal mortality reduction and the prevention of stillbirths were endorsed by 194 member states as part of the Every Newborn Action Plan (ENAP) [6, 7], resulting in the first ever global target for neonatal mortality in the SDGs (SDG3.2) [8]. To track progress towards these targets, countries require more reliable and timely data. A multi-partner ENAP Measurement Improvement Roadmap, with actions to improve data for outcomes, coverage and quality of care was led by WHO with London School of Hygiene & Tropical Medicine and published in 2015 [9, 10]. One standout priority was the lack of any rigorous comparison of large-scale household surveys of measurement approaches for stillbirths and neonatal deaths. Demographic and Health Surveys (DHS) [11] and UNICEF’s Multiple Indicator Cluster Surveys (MICS) [12] are used every 3 to 5 years in more than 60 countries with standardised tools and reports and provide data on pregnancy outcomes for countries that account for around two thirds of the global burden related to pregnancy outcomes.

## What was done, where and by whom?

**Fig. 1:**
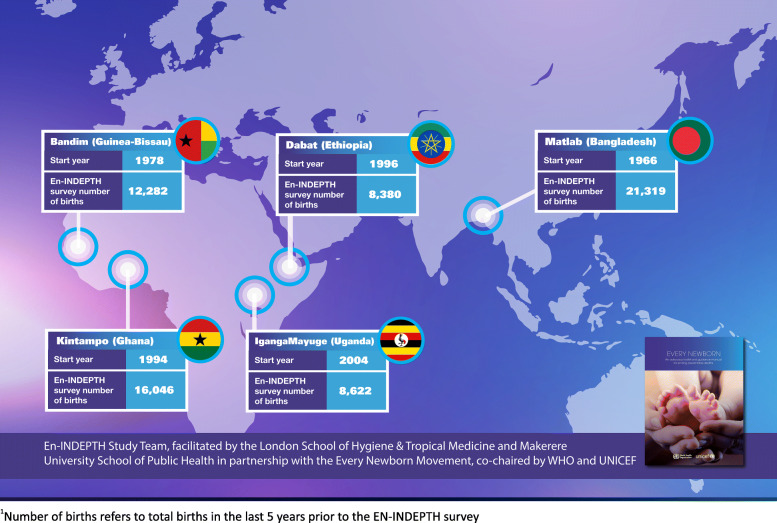
Five EN-INDEPTH study sites in Africa and Asia

The “*Every Newborn-International Network for the Demographic Evaluation of Populations and their Health*” (EN-INDEPTH) study is a direct answer to the gap in counting every newborn, whether alive or stillborn, and aims to improve data, especially for mortality tracking to inform national progress towards SDG3 and ENAP targets. The study’s main aim was a randomised comparison of two approaches in women’s survey reports for capturing stillbirths/neonatal deaths (Full Birth History, or Full Pregnancy History) [13]. In total > 69,000 women were surveyed; in addition to a large quantitative dataset, qualitative data were collected to assess barriers and enablers to reporting pregnancy outcomes in various contexts. The study was set in five health and demographic surveillance system (HDSS) sites within the INDEPTH Network [14]—Bangladesh, Ethiopia, Ghana, Guinea-Bissau and Uganda (Fig. 1). These settings are in ENAP high-burden, priority countries and were selected based on pregnancy surveillance quality criteria after an open call for applications from all INDEPTH sites. Teams from all sites played lead roles in the study, facilitated by a team at London School of Hygiene & Tropical Medicine with Makerere University and funded by Children’s Investment Fund Foundation (CIFF) in support of ENAP.

We are indebted to Professor Peter Byass who was the Senior External Editor for this series until his untimely death in August 2020 [15–17]. This series is dedicated to his memory.

## What can be done now in surveys?

The randomised comparison of survey modules, published in Lancet Global Health [13], found that a Full Pregnancy History approach has potential to increase reporting of stillbirths in high-burden contexts.

**Fig. 2:**
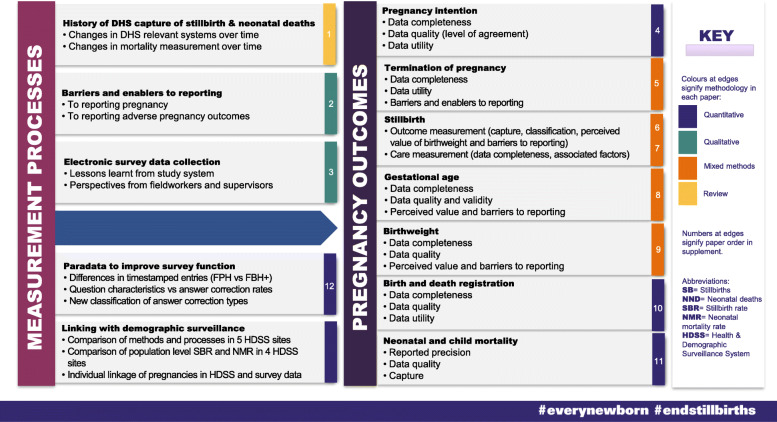
Overview of the 12 papers in the EN-INDEPTH study supplement. More details in matrix of key findings per paper

Our editorial summarises the main findings from the 12 papers in this supplement (Fig. 2). Initial papers cover measurement processes. The first is a detailed review of DHS measurement of pregnancy outcomes over the last four decades and analyses stillbirth and neonatal mortality data, comparing measurement methods by country and over time [18]. The second paper reports on multi-country qualitative research regarding barriers and enablers to reporting of pregnancy outcomes. There are surprisingly similar findings across settings, but the barriers are highest for reporting termination of pregnancy, followed by stillbirths. Fewer barriers were reported for neonatal deaths, although all outcomes involved stigma among affected women [19]. The third paper summarises development and use of the electronic data system used in EN-INDEPTH, including lessons relevant for large-scale surveys transitioning to electronic systems. Importantly, even with a standard tool and software and training guides, there was variation regarding survey implementation especially in one site [20]. Each of the eight pregnancy outcome papers provides more detailed analyses informing survey measurement for that specific outcome, and relevant research questions [21–28] (see matrix of key findings per paper).

The final paper presents novel analyses of paradata, i.e., timestamped records tracking the process of electronic data collection. From 3.6 million timestamped entries from 65,768 interviews, 84% of interviews had at least one corrected answer, giving insights into questions and practices that can be improved to reduce corrections, save time and enhance data quality [29].

Improving survey data is feasible now based on the learning from the EN-INDEPTH study. We are delighted that the new DHS model questionnaire has already changed to a Full Pregnancy History based on the EN-INDEPTH study [13, 30]. Further implications from this study evidence, which are feasible now in surveys, include:

- *Adding novel or adapted questions to better capture pregnancy outcomes.* There are cross-cutting implications from the papers on seven different pregnancy outcomes (see Fig. 2), notably more consistent use of antenatal care and child health cards when conducting surveys. This talks to both training of survey data collectors and campaigns to encourage families to use and value these home-held records. Each paper gives specific improvements to questions possible for each of these outcomes (see matrix of key findings per paper).
- *Removal of skip patterns* that excluded women who had a stillbirth from answering pregnancy care questions. Our data show that these women report effectively [23] and since they are more at risk, excluding them will overlook important complications or outcomes such as caesarean section [24].
- *Adaptation to context* by using local words for stillbirth or preterm birth and considering the cultural and societal barriers to reporting of pregnancies and adverse pregnancy outcomes including termination of pregnancy [19, 22].

## What next in research?

Although we highlight actions feasible now, there are recurrent themes that underline the need for further essential research. We summarise research themes by the steps of survey design and implementation as follows:

- *Data quality for pregnancy outcomes*: Research is required to develop robust data quality assessments for stillbirths and neonatal deaths. Investigation of accuracy, omissions and associated factors may improve child mortality estimation methods. For birthweight, methods to minimise heaping are crucial for data quality [25, 31]. As gestational age questions are included in surveys, systematic approaches to assessing data quality and further improving these questions are important [26, 32].
- *Survey content and structure*: Quantitative analyses of question performance, considering structure and skip patterns would add value, for example using paradata. Such analyses can inform improvement of questions, e.g., by targeting questions with higher correction rates. Linked qualitative assessment of such questions would be valuable given the cost of running these surveys and potential to improve responses, time taken and data quality.
- *Implementation research*: Training methods (including more virtual or hybrid training) and supervision have rarely been systematically studied. The transition to electronic capture also requires more assessment to optimise functionality. The software used in EN-INDEPTH study had many tracking dashboards [20]—for example to compare performance of data collectors—yet these were variably used across sites and further optimisation and use of dashboards in routine DHS or MICS could help operationalise local data collection feedback loops.
- *Linkage to facility data*: Around 80% of the world’s births are in now in health facilities. Improving facility data on key outcomes (e.g., gestational age, birthweight, stillbirth or early neonatal deaths), but to be captured in surveys, requires improved communication to women so that outcomes and care are known. Strengthening investment in facility records provides opportunities to link facility data on content and quality of care to population-based survey data. Achieving this could enable assessment of effective coverage in settings where a sizeable proportion of births occur at home. Currently, estimating effective coverage for maternal/newborn health relies on complex analyses of special datasets [33], so more routine and country-led approaches are needed, including in routine facility systems [34].

## Conclusion

EN-INDEPTH study was an ambitious, multi-country study but is also an example of an equitable partnership with multi-directional learning that offered a host of informal and formal opportunities including three nested PhDs. Given an imperative for decolonising global health, we encourage more examples of initiatives with international multi-directional learning networks. In reality these take time and effort—we call on academic institutions and funding partners to enable such processes and reform structures and funding systems to do so.

EN-INDEPTH study has shown that large-scale surveys can be improved *now* in order to increase data capture and quality for pregnancy outcomes, which will in turn inform the actions of national planning and global investments. Importantly, this data can enable improved coverage, equity and quality of care to save the lives of mothers and babies everywhere. However, improved data alone will not change outcomes—investments in next generation research, programme and policy leadership are critical to improve and apply data in the highest burden settings.

## Data Availability

Not applicable.

## Abbreviations

CIFF: Children’s Investment Fund Foundation
DHS: Demographic and health surveys
ENAP: Every Newborn Action Plan
EN-INDEPTH: Every Newborn-International Network for the Demographic Evaluation of Populations and their Health
HDSS: Health and demographic surveillance system
INDEPTH: International Network for the Demographic Evaluation of Populations and their Health
LMICs: Low- and middle-income countries
LSHTM: London School of Hygiene & Tropical Medicine
MICS: Multiple Indicator Cluster Survey
SDGs: Sustainable Development Goals
UNICEF: United Nations Children’s Fund
WHO: World Health Organization
